# A Modified Balanced Steady State Free Precession Sequence for Overhauser Magnetic Resonance Imaging

**DOI:** 10.1002/mrm.70453

**Published:** 2026-06-07

**Authors:** Kai Buckenmaier, Friedemann Bullinger, Georgiy Alekseevich Solomakha, Marcel Schneider, Jörn Engelmann, Philipp Pohlmann, Pavel Povolni, Laura Kuebler, André Ferreira Martins, Klaus Scheffler

**Affiliations:** ^1^ High‐Field Magnetic Resonance Center Max Planck Institute for Biological Cybernetics Tübingen Germany; ^2^ Werner Siemens Imaging Center, Department of Preclinical Imaging and Radiopharmacy, University Hospital Tübingen Eberhard‐Karls University Tübingen Germany; ^3^ Cluster of Excellence iFIT (EXC 2180) “Image‐Guided and Functionally Instructed Tumor Therapies Eberhard‐Karls University Tübingen Germany; ^4^ German Cancer Consortium (DKTK) and German Cancer Research Center (DKFZ), Partner Site Tübingen Tübingen Germany

**Keywords:** bSSFP, hyperpolarization, OMRI, Overhauser DNP, ULF MRI

## Abstract

**Purpose:**

Low‐ and ultralow‐field magnetic resonance imaging (ULF MRI) have inherently low signal‐to‐noise ratio (SNR) by design. Overhauser dynamic nuclear polarization (ODNP) stands out as an effective solution for continuous signal enhancement. To accelerate imaging and reduce radiofrequency (RF) power deposition—which is critical due to the high‐frequency RF fields required for ODNP—efficient pulse sequences are essential. For instance, a balanced steady‐state free precession (bSSFP) sequence offers high signal efficiency. However, conventional bSSFP sequences are susceptible to *B*_0_ inhomogeneities and temporal field drifts, which can lead to banding artifacts.

**Methods:**

In this work, we mitigated the limitations of banding artifacts by developing a modified bSSFP sequence (bSSFP180) that allows interleaved polarization and acquisition, thereby tailoring it for ODNP‐enhanced ULF MRI. The unmodified and modified sequences are compared in an imaging experiment.

**Results:**

The proposed sequence effectively suppresses banding artifacts and demonstrates robust performance under inhomogeneous field conditions. Experimental MRI results demonstrated that bSSFP 180 achieves banding artifact‐free performance.

**Conclusion:**

The banding artifact‐free bSSFP180 images represent a significant advancement toward the practical and reliable use of ODNP‐enhanced imaging in future biomedical applications. This is particularly relevant for MRI experiments that employ lightweight, cost‐efficient, permanent magnet systems in low‐field environments, which often face challenges related to field instability and inhomogeneity.

## 1 Introduction

Magnetic resonance imaging (MRI) has undergone significant advancements since its inception, achieving unprecedented resolution, contrast, and diagnostic precision. Three main factors have mainly driven this progress: the use of higher magnetic field strengths [1], the development of more efficient magnetic resonance imaging (MRI) sequences [2], and the capability to hyperpolarize heteronuclear spin states, enhancing contrast by several orders of magnitude [3]. In terms of sequence development, MRI offers a broad range of acquisition strategies, making it a highly versatile diagnostic tool [4]. Notably, the balanced steady‐state free precession (bSSFP) sequence delivers exceptional imaging speed and signal‐to‐noise efficiency [5, 6]. The bSSFP sequence is a widely used MRI technique that finds application in relevant medical fields, including cardiology, neurology, oncology, and pulmonology [5].

The principles of bSSFP, which are based on establishing a steady state through gradient balancing, are well established [6]. Image quality depends critically on field homogeneity, susceptibility‐induced frequency shifts, [7] and variations in the *B*_1_ radiofrequency (RF) field [8] that excite the magnetization. These contributions can generate artifacts, such as banding patterns, signal voids, and non‐uniform contrast. Those artifacts can be estimated and analyzed using Bloch‐equation‐based simulations [9], which are a powerful tool for modeling and mitigating bSSFP artifacts, as well as optimizing sequence design, which has, for example, led to improved applications in functional MRI [10].

Another approach to improve signal intensity is hyperpolarization [11, 12, 13, 14]. The advantage of hyperpolarized MRI is that the signal intensity becomes independent of the thermal spin polarization due to the static magnetic field strength *B*_0_. The majority of hyperpolarization methods involve quickly transporting a small bolus that has been hyperpolarized in a different device to the MRI scanner while it remains hyperpolarized. Hyperpolarization techniques, such as dissolution dynamic nuclear polarization (dDNP) [15, 16], parahydrogen‐induced polarization (PHIP) [17] and the non‐hydrogenative signal amplification by reversible exchange (SABRE), are used in this context [18]. ^13^C‐labeled compounds are most commonly used in hyperpolarized MR experiments because their quaternary‐bound carbons exhibit long longitudinal relaxation times (*T*_1_) of up to several minutes [18, 19], enabling polarization levels exceeding 20% [19, 20]. This high degree of hyperpolarization has allowed extensive in vivo studies and even translation to clinical applications [14, 21, 22]. One of the most promising applications of hyperpolarized MRI in translational medicine is imaging tumor metabolism via the Warburg effect using hyperpolarized ^13^C‐labeled pyruvate. This technique is approaching clinical translation and shows considerable potential for future diagnostic applications [23, 24]. Most magnetic resonance spectroscopic imaging (MRSI) approaches, however, rely on chemical shift imaging techniques that, while providing rapid spectral acquisition, suffer from limited spatial resolution and signal loss due to repeated perturbation of the ^13^C magnetization. In contrast, the bSSFP sequence offers the potential for rapid, high signal‐to‐noise ratio (SNR), and extended acquisitions by efficiently preserving magnetization and maintaining polarization over time [25, 26, 27].

A further technique that uses hyperpolarization is Overhauser MRI (OMRI). With OMRI, the sample is hyperpolarized directly within the MRI system [28, 29, 30, 31, 32, 33]. This technique utilizes the Overhauser dynamic nuclear polarization (ODNP) effect [34], which transfers spin order from electrons to protons by utilizing an RF field (RF_ODNP_) at the electron Larmor frequency. The electron Larmor frequency is approximately 660 times higher than the proton Larmor frequency, resulting in electrons being 660 times more polarized than protons. Depending on whether the coupling mechanism between the unbound electrons and the target protons is scalar or dipolar, the target protons are hyperpolarized either in phase or out of phase with respect to thermal equilibrium polarization [35]. While ODNP achieves enhancement factors in the low 100 range, which are lower than those of other hyperpolarization techniques such as dDNP or PHIP, it allows sample protons at much higher concentrations to be continuously hyperpolarized in the investigated tissue. A key limitation of OMRI is its dependence on millimolar concentrations of unbound electrons, which are typically provided by exogenous radicals, as natural radical concentrations are too low to sustain the effect [36]. The use of various free radicals, such as nitroxide‐based radicals (2,2,6,6‐Tetramethylpiperidinyloxyl, TEMPO and its derivatives; 3‐Carboxy‐PROXYL, CP) or trityl radicals (OXO64) has been demonstrated in in vivo OMRI experiments in the low field (LF) range up to 0.2 T [29, 32, 37]. These OMRI approaches have been used to investigate physiological parameters in small‐animal models (rats and mice), including pO_2_ level [37, 38, 39], tissue redox status, [40, 41, 42] and pH value [43, 44, 45]. However, OMRI applications remain largely limited to these models to date due to constraints relating to the specific absorption rate (SAR) and the limited penetration depth of the RF_ODNP_ excitation field.

Developing suitable, efficient, and non‐toxic radicals is an ongoing challenge that creates new opportunities for tailoring and functionalizing radical species [36, 46]. At higher fields (*B*_0_ > 25 mT), the penetration depth of the required RF_ODNP_ field decreases due to the increase in frequency above the GHz range, making the investigation of larger sample volumes difficult (the penetration depth in tissue at 1 T, which corresponds to ≈28 GHz, is ≈1 mm) [47]. Additionally, the SAR of RF_ODNP_ increases with higher RF frequencies. This presents a significant challenge, though the proton Larmor frequencies below 1 T are still low enough to avoid SAR issues. Therefore, the optimal operating range for OMRI is in the low‐field [28, 31, 32, 33, 38, 48, 49, 50, 51, 52] and ultralow‐field (ULF, *B*_0_ < 10 mT) regime [29, 30, 52, 53]. We hypothesize that combining a modified bSSFP sequence that incorporates the Overhauser effect—first introduced by Sarracanie et al. on a 6 mT MRI system [54]—with a superconducting quantum interference device (SQUID)‐based ULF MRI setup will enable efficient proton signal enhancement and artifact‐free imaging at substantially lower magnetic fields. This would overcome the sensitivity and field inhomogeneity limitations inherent to conventional OMRI.

In this paper, we demonstrate for the first time the use of a bSSFP sequence in combination with a SQUID‐based ULF MRI system at *B*_0_ ≈ 850 μT (proton Larmor frequency ≈ 36.2 kHz) [30], where the signal was enhanced via the Overhauser effect. Additionally, we have introduced the bSSFP180 sequence to eliminate banding artifacts, thereby significantly improving image quality.

## 2 Methods

### 2.1 Sequences

BSSFP sequences are widely recognized for their high SNR efficiency and ability to generate *T*_2_/*T*_1_‐weighted contrast, making them highly attractive for fast imaging applications, with *T*_2_ being the transverse relaxation time. However, banding artifacts are a well‐known limitation of bSSFP—periodic signal voids that appear as dark bands in the image. These arise from off‐resonance effects due to magnetic field inhomogeneities, where resonance frequency deviations of odd integer multiples of 1/(2·TR) result in destructive interference and local signal cancelation, with TR being the repetition time of the sequence. This sensitivity requires precise field shimming, although the artifacts are less pronounced in low‐field MRI due to reduced frequency dispersion.

**FIGURE 1:**
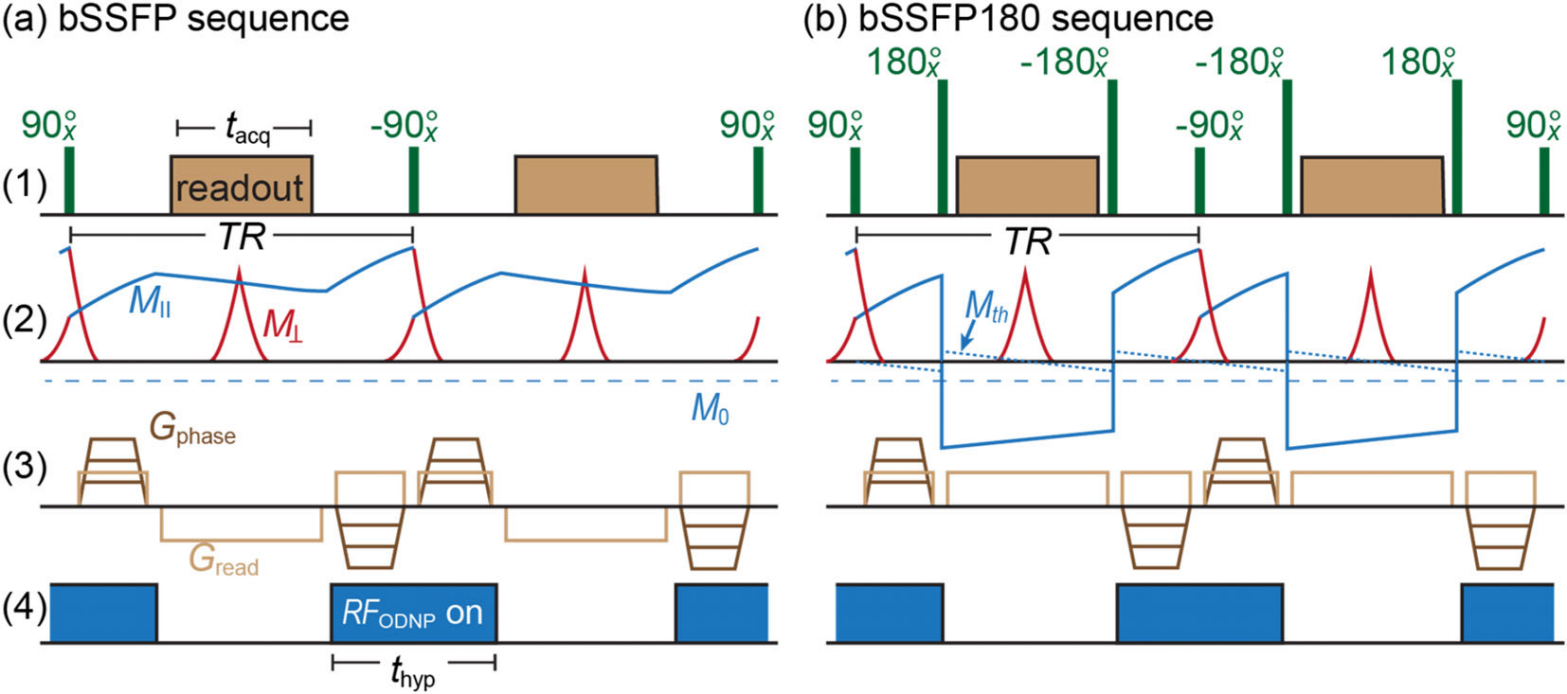
MRI sequences: Schematic of the conventional (a) and modified (b) bSSFP sequences for ODNP. Line (1) marks the positions of the 90° and 180° *B*_1_ pulses (green rectangles) as well as the readout periods. Line (2) marks the longitudinal (blue line) and transversal (red line) magnetization as well as the thermal equilibrium magnetization, *M*_0_ (blue dashed line) and the thermal magnetization without hyperpolarization, *M*_th_ (blue dotted line). The 90° pulses flip *M*_II_ to *M*_⊥_ (the red line changes to the blue line), and vice versa. The 180° pulses reverse the sign of *M*_II_ and *M*_⊥_. Line (3) illustrates the phase *G*_phase_ and read *G*_read_ gradient. For clarity, *G*_read_ is distinguished from *G*_phase_ by its rectangular and trapezoidal shapes, respectively. Line (4) shows the hyperpolarization period *t*_hyp_ when RF_ODNP_ is switched on. The black lines indicate zero value.

A typical bSSFP sequence with alternating excitation adapted for ODNP experiments is shown in Figure 1a [29, 54]. This sequence includes an additional RF_ODNP_ pulse with rectangular envelope. The RF_ODNP_ pulse is in resonance with the electron Larmor frequency and is active during phase encoding and the *b*_1_ pulse. During the RF_ODNP_ pulse the sample is hyperpolarized, defining the hyperpolarization period *t*_hyp_ (indicated by the longitudinal sample magnetization *M*_II_ build‐up along the *z‐*direction, blue line in Figure 1a). The frequency of the RF_ODNP_ pulse is set to ≈90 MHz at 850 μT in order to excite the *T*_16π_ transition of the two‐spin system formed by the unbound electron spin and the ^14^N nucleus of the TEMPO radicals used in this experiment [55]. Active gradients during the hyperpolarization phase restrict the on‐resonance condition to a single slice within the RF_ODNP_ field's excitation bandwidth. This could be problematic for larger samples.

Note that the sample is hyperpolarized with an inverse sign relative to the thermal magnetization, because of the dipolar coupling between protons and the used nitroxide radicals (blue dashed line) [56]. When the RF_ODNP_ pulse is turned off, the longitudinal magnetization decays toward the thermal equilibrium magnetization. After several TRs, the sequence reaches a steady state, producing an echo at 0.5 TR (see transverse magnetization *M*_⊥_ within the *xy*‐plane, red line in Figure 1a). As for conventional bSSFP without ODNP, the echo amplitude is sensitive to field inhomogeneities.

To overcome this sensitivity, the sequence can be modified by adding two 180‐degree pulses (*B*_1_) at 0.25 TR and 0.75 TR (Figure 1b), referred to as the “bSSFP180” sequence in the following discussion. Due to the 180‐degree pulses, the read gradient is not inverted as in conventional bSSFP sequences. The first 180‐degree pulse produces a spin echo at 0.5 TR, and the second 180‐degree pulse produces a spin echo at TR, which is then flipped back to longitudinal magnetization by the 90‐degree *B*_1_ pulse. This sequence has some similarities to the Driven Equilibrium Fourier Transform (DEFT) technique [57, 58]. However, the 90–180–180–90 block in DEFT is repeated sequentially with a certain delay between consecutive blocks, whereas in bSSFP180 these blocks are repeated instantaneously as the last 90‐degree flipback pulse is also used as an excitation pulse. Thus, the bSSFP180 pulse train has one 90‐degree pulse less and thus a different signal behavior than DEFT. The steady state signal of bSSFP180 without hyperpolarization is actually close to zero in contrast to the DEFT sequence.

Adding the 180‐degree pulses has several advantages. The creation of a spin echo allows the signal to decay with the relaxation time *T*_2_, unlike the standard ODNP bSSFP sequence, where the signal critically depends on field inhomogeneity, allowing for longer TRs. This is particularly beneficial for ULF MRI, where longer acquisition, gradient, and pulse times result in slower sequences. In addition, the signal decays more slowly during readout because the difference between the thermal equilibrium magnetization and the sample magnetization is slightly smaller (the blue dashed line and blue line are on the same side). Other advantages, such as the absence of banding artifacts, are discussed below.

As mentioned above, this modified bSSFP sequence can only be used in combination with hyperpolarization. Without hyperpolarization, the two 180‐degree pulses prevent the sample from building up thermal magnetization *M*_th_ (here, “thermal” implies that the magnetization relaxes toward thermal equilibrium). The longitudinal magnetization that accumulates before the first 180‐degree pulse is inverted and reaches almost the same value before the second 180‐degree pulse as it had before the first. After inversion, the signal decays again, reaching nearly zero after 1 TR (Figure 1b, *M*_th_ blue dotted line).

In conventional bSSFP sequences without hyperpolarization, the maximum achievable transverse magnetization *M*_⊥,max_ corresponds to 0.5 *M*_0_ [6], where *M*_0_ denotes the thermal equilibrium magnetization of the sample. However, this maximum magnetization can only be achieved for relaxation times of *T*_1_ = *T*_2_. This condition is usually almost fulfilled at ULF. For a 2 mM solution of TEMPO in water, we determined relaxation times experimentally, as described in [36], and found that *T*_1_ = (616 ± 15) ms and *T*_2_ = (575 ± 2) ms. When hyperpolarization is applied, this expression is modified to *M*_⊥,max_ = 0.5 · *M*_0_ · *E*_max_ · *D*_hyp_, with *E*_max_ representing the maximum achievable signal enhancement for the given sample and *RF*_ODNP_ power (enhancement reached after irradiating the sample for an infinite long time) [36, 59], and *D*_hyp_ denoting the duty cycle of the RF field—that is, the fraction of the sequence period during which hyperpolarization is active (*D*_hyp_ = *t*_hyp_/TR). Notably, for the bSSFP180 sequence presented below, the maximum *D*_hyp_ is limited to 0.5 due to sequence timing constraints (the time between the two 180‐degree pulses), whereas in the conventional bSSFP sequence, *D*_hyp_ is only constrained by the duration of the data acquisition window (*t*_acq_) and can exceed 0.5.

In theory, a fundamental requirement of the bSSFP sequence is that the integrated gradient over a TR should be zero in order to prevent phase accumulation and signal dephasing caused by gradient fields. However, in the bSSFP180 sequence, this condition appears to be initially transgressed as the readout gradient *G*_read_ is not alternated in polarity, unlike the phase encoding gradient *G*_phase_, which follows an alternating pattern (see Figure 1). Nevertheless, the inclusion of a 180‐degree refocusing *B*_1_ pulse effectively inverts the magnetization, thereby reversing the accumulated phase from any preceding gradients. Consequently, all gradients occurring between two successive 180‐degree pulses must be considered with flipped signs. Taking this effect into account makes it clear that the cumulative moment of the readout gradient is effectively zero, thus satisfying the dephasing cancelation condition required for bSSFP signal stability. To further enhance sequence stability, a 180‐degree phase shift is applied to all *B*_1_ pulses following each TR. This prevents the buildup of phase errors caused by imperfect 180‐degree refocusing pulses, which would otherwise result in center line or mirror image artifacts similar to those seen in multi‐spin echo sequences [60].

The sequence did not use slice selection gradients. Therefore, the 2D version of the sequence projects the entire sample onto a single slice. Non‐selective 5‐ms‐long *B*_1_ pulses with a sinc function envelope were generally applied.

The images in the results section were acquired using the parameters shown in Table 1. The time domain of the transverse and longitudinal magnetization in the transient state is qualitatively plotted in Figures 1 and S1. We processed the acquired data as described in the Supporting Information.

**TABLE 1:** Sequence parameters for the 2D (Figure 3) and 3D images (Figure 6). Additionally, a zero‐padding factor of 1 was used for reconstruction of all images.

Figure	Figure 3a	Figure 3b	Figure 3c	Figure 3d	Figure 6
*t*_hyp_ [ms]	43	202	42	193	93
*t*_acq_ [ms]	28.5	190	40.0	178.5	77
Averages	15	4	15	4	32
Phase steps	13	13	13	13	13 × 13
TR [ms]	100	400	100	400	200
*T*_total_	19.5 s	20.8 s	19.5 s	20.8 s	9 min 1 s
*G*_read_ res. [mm]	2	2	2	2	1.8
*G*_phase_ res. [mm]	2	2	2	2	1.8 × 1.8

The effect of banding artifacts was illustrated by measuring the steady‐state MR magnetization *M*_⊥_ as a function of the resonance frequency offset, while keeping the *B*_1_ pulse frequency fixed and gradients turned off. The frequency offset was expressed as offset angle *ϕ* accumulated during the TR between successive 90° pulses. This was implemented experimentally by assigning the offset angle as the phase of the 90° pulse. The resulting bSSFP profiles show the dependency of the steady state amplitude as a function of the offset angle *ϕ*. A critical factor in obtaining reliable experimental bSSFP profiles is the minimization of magnetic field inhomogeneities. To address this, we applied first‐order shimming using the typical gradient coils used for imaging.

The profiles in the results section were acquired using the parameters shown in Table 2 for the bSSFP profiles and in Table 3 for the bSSFP180 profiles. Averaging was achieved by sweeping the phase over five complete periods and summing the resulting signals of these periods in post‐processing.

**TABLE 2:** bSSFP sequence parameters for Figures 4a and S5.

Color	Cyan	Blue	Purple	Red	Orange	Yellow
*t*_hyp_ [ms]	52.5	65	77.5	102.5	152.5	202.5
*t*_acq_ [ms]	40	52	65	89.5	139	188.5
Averages	5	5	5	5	5	5
TR [ms]	100	125	150	200	300	400

**TABLE 3:** bSSFP180 sequence parameters for Figure 4b.

Color	Cyan	Blue	Purple	Red	Orange	Yellow
*t*_hyp_ [ms]	43	55.5	68	93	143	193
*t*_acq_ [ms]	28.5	41	53	78	128	178
Averages	5	5	5	5	5	5
TR [ms]	100	125	150	200	300	400

### 2.2 Simulations

The simulations of the bSSFP and bSSFP180 signal amplitudes as a function of the local off‐resonance frequency were performed using the Bloch equations in the rotating frame (see Supporting Information for a detailed description of the simulations). The time dependency of an ensemble of spins (500 spins) was simulated over the period of several TRs until a steady state was reached. The summed‐up magnetization of all spins was taken at TR/2 and used for the simulated transverse magnetization *M*_⊥_. Relaxation times were measured experimentally and incorporated into the simulations (see Table S1 in Supporting Information for simulation parameters).

To incorporate *B*_0_ inhomogeneity into the simulation, a Larmor frequency offset was assigned to every simulated spin of the ensemble. This frequency offset from the Larmor frequency was randomly distributed according to a normal distribution. The standard deviation *σ* of the distribution was chosen as the measure of *B*_0_ inhomogeneity.

### 2.3 Hardware and Phantoms

**FIGURE 2:**
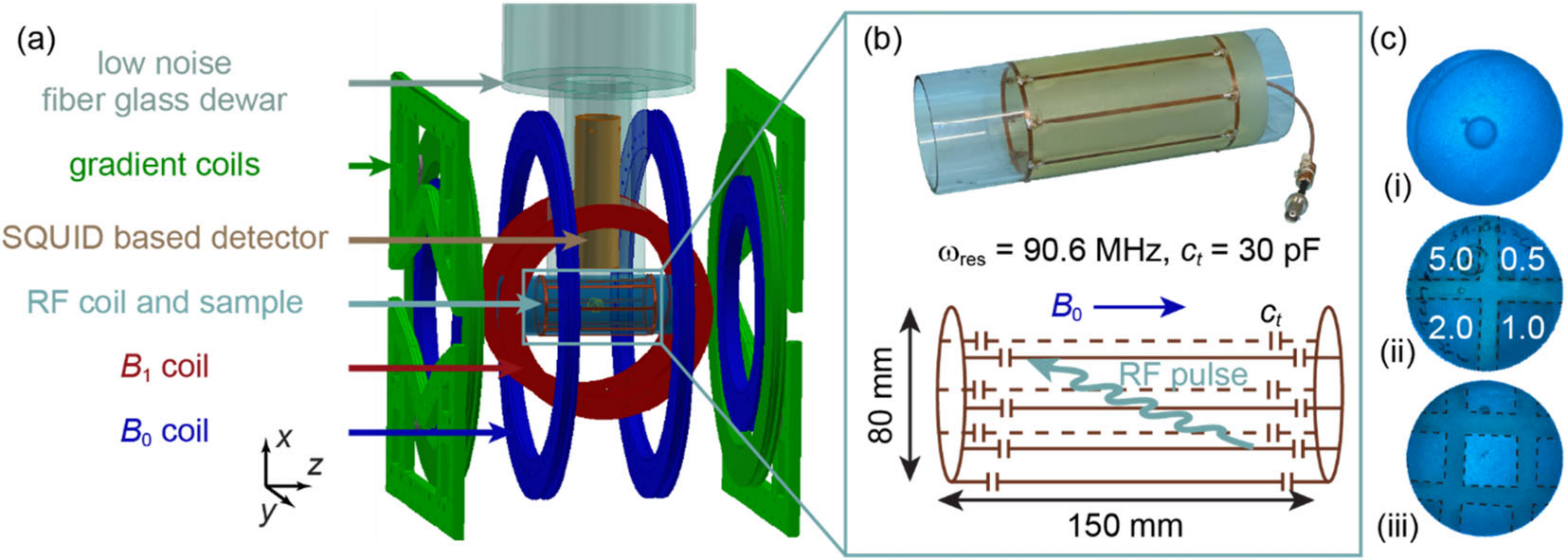
Experimental setup: (a) Schematic of the SQUID‐based ULF MRI setup. (b) Photo and schematic of the RF_ODNP_ low‐pass birdcage coil used for hyperpolarization via the ODNP effect. (c) Photos of the phantoms used in this study, with chamber walls highlighted by dashed lines for clarity. The white numbers on phantom (ii) indicate the TEMPO concentration in mM.

The ULF MRI setup (Figure 2a) uses a SQUID‐based magnetic field sensor to detect the transverse magnetization and replaces the receiver Faraday coils used in high‐field MRI devices. During signal readout of length *t*_acq_, all coils except the *B*_0_ and *G*_read_ coils were galvanically isolated from the current amplifiers to minimize noise. The static *B*_0_ field is generated by a tetra coil, an extended Helmholtz‐like coil consisting of four coil elements. A *B*_1_ Helmholtz coil is used to provide spin excitation. Three sets of gradient coils perform spatial encoding of the MR signal: two sets of planar gradient coils (*G*_x_ and *G*_y_ gradients, only one set is shown in Figure 2a for clarity) and one Maxwell coil (*G*_z_ gradient). The entire setup is located in a magnetic and electrical shielding chamber with a residual field of < 10 nT, as reported previously [30].

The phantoms used in this study were placed in an RF_ODNP_ low‐pass birdcage coil [48], which is used for hyperpolarization via the Overhauser effect (Figure 2b). It is tuned to *f* = 90.6 MHz and matched to *Z* = 50 Ω to minimize reflections from the power amplifier. The *Q*‐factor of the loaded coil is *Q*_unloaded_ = 148 and *Q*_loaded_ = 171 for the unloaded coil, measured with a network analyzer [29]. The RF_ODNP_ pulse is generated by a synthesizer (HAMEG HM8134‐3) and amplified by a broadband RF amplifier (Frankonia FLH‐50A). The absorbed power of the phantom *P*_abs_ = 4 W was determined using $P_{\mathrm{abs}}=P_{\text{in}}\frac{Q_{\text{unloaded}}-Q_{\text{loaded}}}{Q_{\text{unloaded}}}$ with the amplifier input power *P*_in_ ≈ 30 W.

The three different phantoms used in this study share the same outer size and inner volume of *V* ≈ 11 mL, but differ in their internal structure (Figure 2c): Phantom (i) was filled with 2 mM TEMPO (chemical structure in Figure S2) dissolved in Phosphate Buffered Saline (PBS). Since each TEMPO molecule provides one unbound electron, the concentration of unbound electrons is similar to the concentration of TEMPO. For phantom (ii), the inner volume is divided into four equally sized chambers, with different TEMPO concentrations (0.5, 1, 2, and 5 mM). For phantom (iii), the inner volume is divided into nine interconnected chambers, all filled with 2 mM TEMPO dissolved in PBS.

## 3 Results

### 3.1 2D Images

**FIGURE 3:**
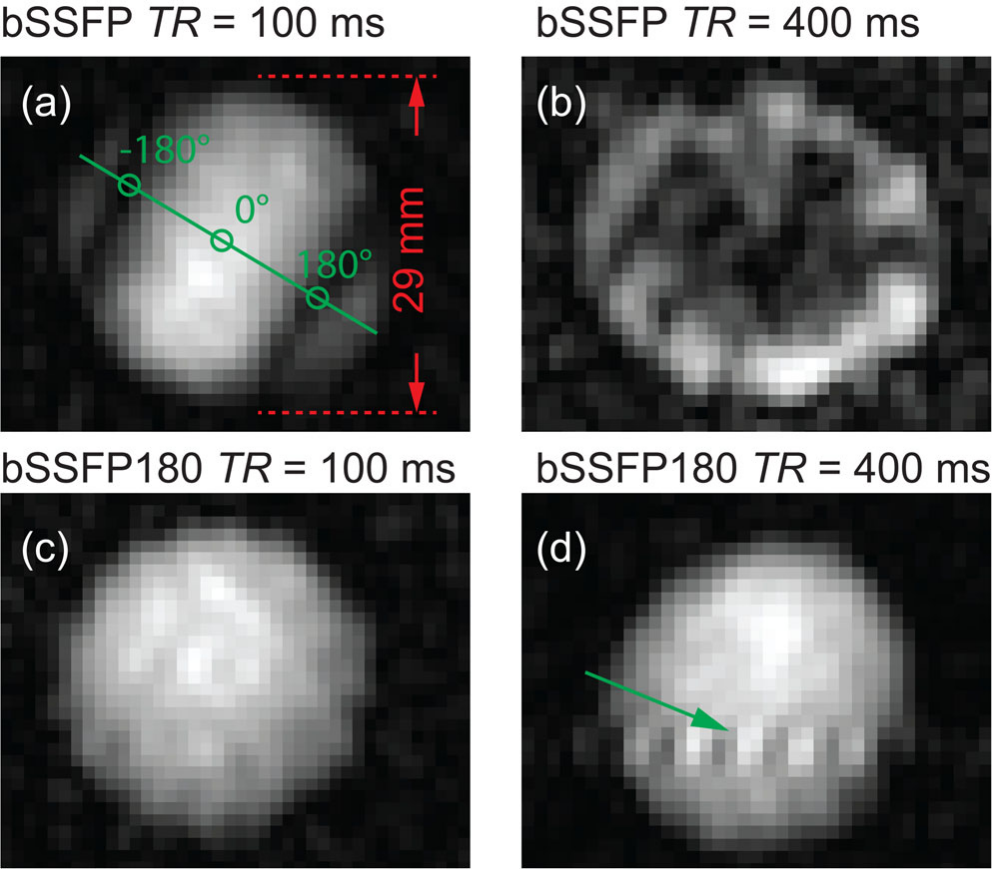
2D images acquired at 850 μT (RF_ODNP_ frequency ≈ 90 MHz) of phantom (i) for different sequence parameters. Images taken with bSSFP exhibit banding artifacts at phase offsets of n·360° (TR = 100 ms, green line with circles), which result in significant signal loss at TR = 400 ms. The spherical phantom shape appears clearly using the bSSFP180 sequence. For TR = 400 ms, a center line artifact appeared at the position of the green arrow. Sequence parameters are listed in Table 1.

To investigate the effect of banding artifacts in the ULF regime, we have acquired test images at *B*_0_ ≈ 850 μT using phantom (i) and the sequence parameters listed in Table 1. The total acquisition time of the images was kept at approximately 20 s to ensure that the SNR of all 2D images (bSSFP and bSSFP180 images) was comparable. Additionally, the resolution in the phase and read directions (*G*_phase_ res. and *G*_read_ res.) was 2 mm for all 2D images. We selected a flip angle of 90° to maximize signal intensity (according to Ref. [6] as the ratio *T*_2_/*T*_1_ is close to unity at ULF) [61]. The influence of TR on banding artifacts was evaluated by comparing bSSFP and bSSFP180 sequences at TR values of 100 and 400 ms (Figure 3). The relatively long TRs in our experiments originate primarily from technical and physical constraints inherent to the ULF MRI setup. During signal acquisition *t*_acq_ with the SQUID‐based detection system, components that could introduce noise must be heavily filtered, such as the *B*_0_ current source and the readout gradient amplifier, or completely galvanically isolated from the experiment using relays. The used relays have switching times of 1–10 ms, which extends the overall sequence duration. This is particularly true for the phase‐encoding gradients and the *RF*_ODNP_ amplifier used for ODNP (as illustrated schematically in Figure 1, line 3, there is a delay between gradient switching) [30]. In addition to these technical limitations, physical constraints also play a significant role. Strong frequency encoding gradients cannot be applied because they introduce noise that scales with their strength. Consequently, long acquisition windows are required to achieve adequate spectral resolution in the readout direction. Concomitant gradients play no observable role in this regime. Noise‐removal techniques, such as EDITOR [62], have the potential to significantly improve image quality by reducing noise from the gradient amplifiers. However, implementing them requires additional detectors for independent noise acquisition. In our case, this would necessitate a second SQUID‐based detector, which would require an additional or modified helium dewar.

At shorter TR (Figure 3a), clear banding artifacts were observed for the bSSFP sequence. Even so, the artifacts resemble a linear sweep of frequencies, which is usually caused by an applied linear gradient. However, no such gradient was applied. Instead, the position of the banding artifacts reflects the intrinsic inhomogeneity of the *B*_0_ coil's static field and the shielding chamber. This differs from the simulated *B*_0_ field inhomogeneity map for the *B*_0_ tetra coil shown in the Supporting Information (Figure S3). As TR increased, the distance between the bands decreased, culminating in widespread signal loss across the sample at TR = 400 ms (Figure 3b). This is consistent with the expected off‐resonance behavior of bSSFP sequences, as discussed in the simulated and measured bSSFP profiles below.

The bSSFP180 sequence shows no visible banding artifacts regardless of the repetition time TR (Figure 3c,d). This robustness is due to the spin‐echo nature of the sequence: the inclusion of a 180° refocusing pulse effectively compensates for static magnetic field inhomogeneities. As a result, off‐resonance effects that would otherwise lead to destructive interference and signal gaps in conventional bSSFP are largely eliminated, thereby preserving image homogeneity even in the presence of field inhomogeneities. We observed that prolonging TR increases the effects of center line artifacts (Figure 3d, green arrow), which arise from non‐perfect *B*_1_‐pulses [60].

### 3.2 bSSFP Profiles: Measurement Versus Simulation

**FIGURE 4:**
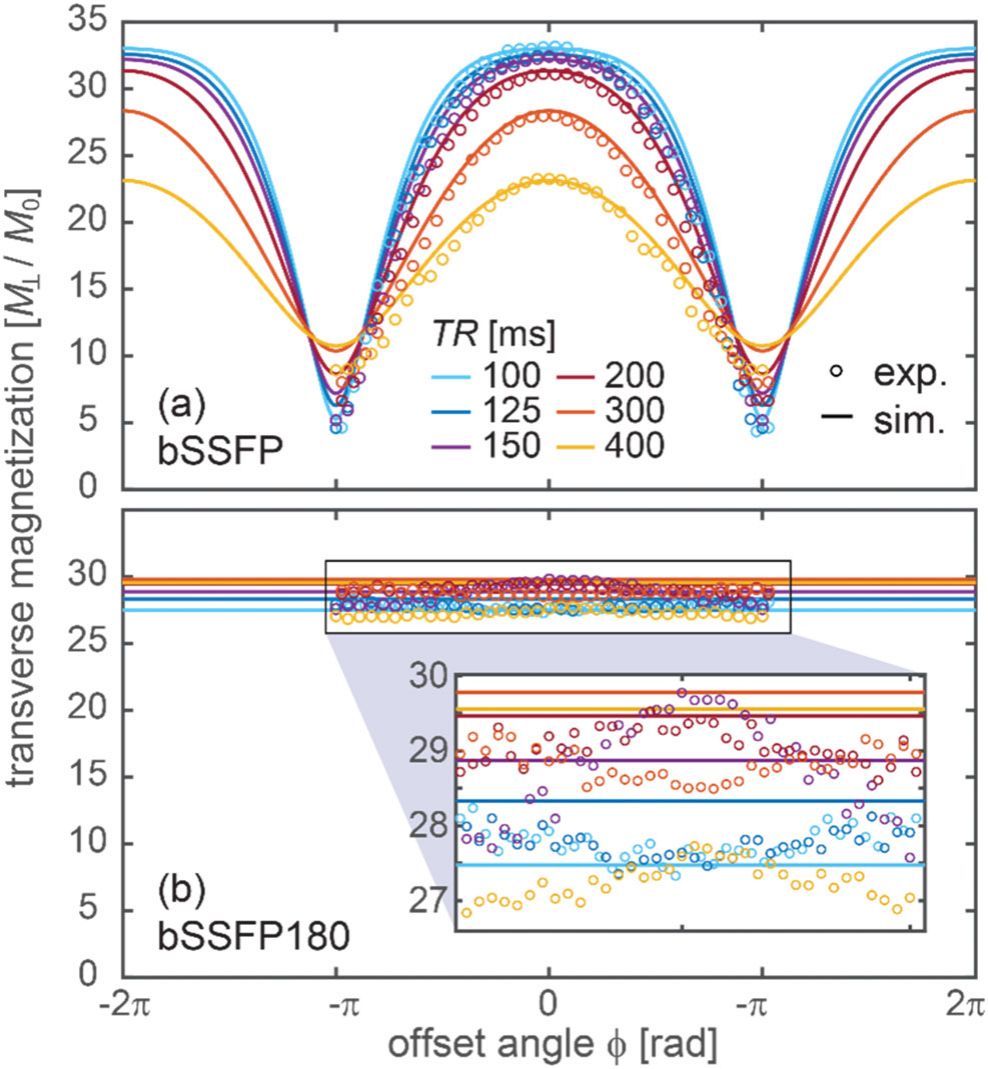
Simulated and measured bSSFP profiles. The transverse magnetization *M*_⊥_ is shown as a function of the offset angle *ϕ* in the steady state for (a) the bSSFP and (b) the bSSFP180 sequence [phantom (i)]. The 180‐degree *B*_1_ pulses have a refocusing effect, causing the signal to be *ϕ* independent. However, the experimental data showed a small dependency on *ϕ* because of non‐perfect *B*_1_ pulses.

To explain the appearance of banding artifacts within the bSSFP sequence and the absence of banding artifacts in the bSSFP180 sequence, we performed simulations and experiments to determine one‐dimensional bSSFP profiles. As expected, the simulated bSSFP profile for the ODNP‐adapted bSSFP sequence (Figure 4a), which mimics the steady‐state magnetization *M*_⊥_ of the green line in Figure 3a, shows the characteristic banding pattern known from conventional bSSFP sequences. The steady‐state signal partially collapses at offset angles of *ϕ* = (2*n*−1)π with *n* being an integer number [6]. The magnetization was normalized to the thermal equilibrium magnetization *M*_0_.

**FIGURE 5:**
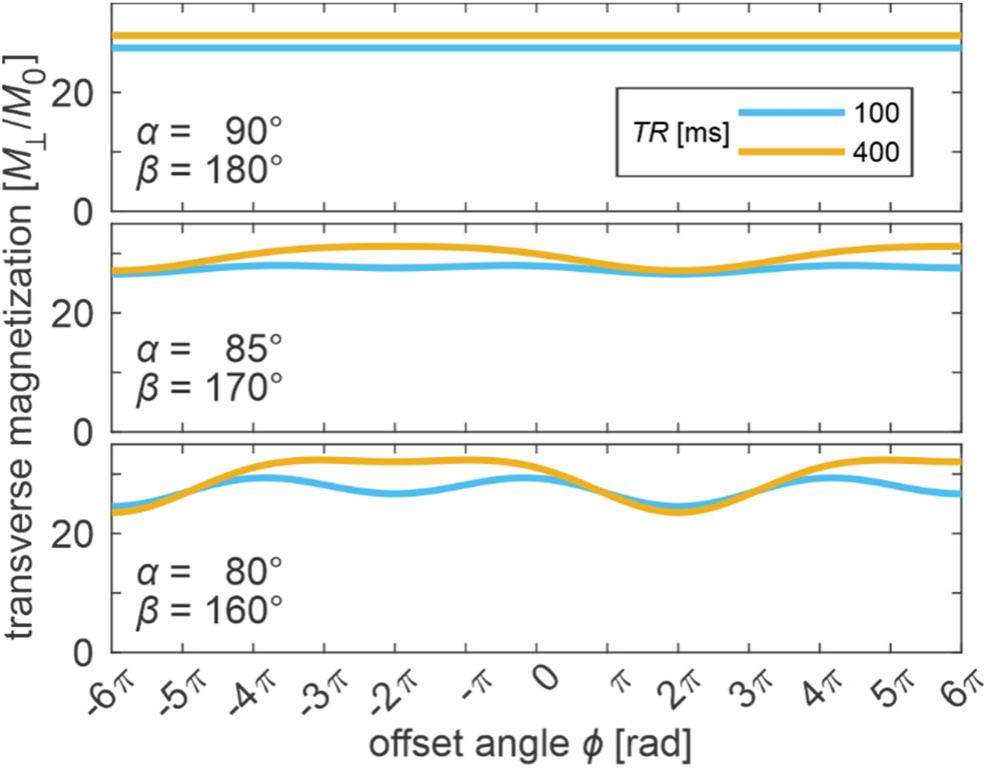
Simulated bSSFP180 profile for *B*_1_‐pulses with flip angles *α* and *β*. Signal loss is visible in certain offset angle regions for growing angle differences to perfect 90°/180° pulses. Detailed simulation parameters are given in Table S1.

In the simulations, *E*_max_ and *σ* were fitted to the measurement data. *E*_max_ was measured by comparing the thermal equilibrium spectrum to a hyperpolarized one (Figure S4). To do so, a simple FID sequence was applied after a long (4*T*_1_) hyperpolarization phase. The resulting fit parameter *E*_max_ = −126, used for the simulations in Figures 4 and 5, is reasonable compared to the experimentally determined *E*_max_ = −136. A previous study found *E*_max_ = −152.7 for infinite RF_ODNP_ power, showing that our fit parameter is realistic [36]. Further details about *E*_max_ can be found in the Supporting Information. The resulting fit parameter for *σ* was 18.51 ppm (15.73 nT).

Figure 4 shows that experimental and simulated results of the bSSFP sequence show qualitatively similar behavior for different TR. Although TR is in the order of hundreds of milliseconds, the profile is symmetric (“pure”), as predicted earlier for low‐field scanners [63].

In comparison, the experimental and simulated bSSFP180 profiles (Figure 4b) show no dependence on the accumulated phase offset. This is expected because the additional 180° *B*_1_ pulses effectively refocus any phase shifts caused by off‐resonance effects (imperfect 180° pulses result in signal loss, see below). However, the steady‐state signal amplitude shows a slight dependence on TR. We have reproduced this trend qualitatively in simulations. Interestingly, the signal amplitude initially increases with TR but decreases for TR = 400 ms. This effect is more pronounced in the experimental data than in the simulations (see the inset in Figure 4b). The simulations did not account for diffusion effects. Diffusion becomes more significant for longer TR*s*, which correlates with longer echo times TEs for the bSSFP180 sequence [64]. This effect likely accounts for the larger signal decay at TR = 400 ms observed experimentally compared with the simulations. Nonetheless, under the specific parameters of this study, its influence is negligible and only apparent in the enlarged view in Figure 4b.

Furthermore, the experimental data showed a minor residual phase dependence (Figure 4b, inset), which can be attributed to imperfections of the 90° and 180° *B*_1_ pulses.

The effect of different *B*_0_ inhomogeneity distributions on the bSSFP profile is plotted in Figure S5 and discussed in more detail in the Supporting Information. The effects of such inhomogeneities are well understood and have been discussed in the literature [7].

### 3.3 Influence of Imperfect *B*_1_ ‐Pulses on the bSSFP180 Sequence

At ULF, the wavelength of the applied *B*_1_ pulses is much larger than the dimensions of typical samples, even those on the scale of the human body. Consequently, spatial variations of the *B*_1_ field across the sample volume are minimal in the ULF regime. This behavior contrasts sharply with that of high‐ and ultrahigh‐field MRI, in which the *B*_1_ wavelength often becomes comparable to or smaller than the sample dimensions. This leads to pronounced field inhomogeneities [1].

Nevertheless, even in the ULF regime, the flip angles in experiments are never perfect. The *B*_1_ flip angle *β* (labeled as the 180° pulse in Figure 1) was determined experimentally by varying the *B*_1_ amplitude within a simple FID sequence, which was performed after an ODNP hyperpolarization period. Furthermore, the 90°‐pulse in Figure 1 was set to $\alpha =\frac{1}{2}\beta$. To illustrate the effect of imperfect 90°‐ and 180°‐pulses on the bSSFP180 sequence, simulations were performed using smaller flip angles. Figure 5 shows that the phase profile for deviating *B*_1_ flip angles result in transverse magnetization loss over certain offset angle regions. This loss is periodic over 8π rather than 2π. This is because of the additional two 180°‐pulses at position 1/4 TR and 3/4 TR increasing the periodicity of the sequence by a factor of 4 in comparison to a standard bSSFP sequence.

### 3.4 bSSFP180 3D Image

**FIGURE 6:**
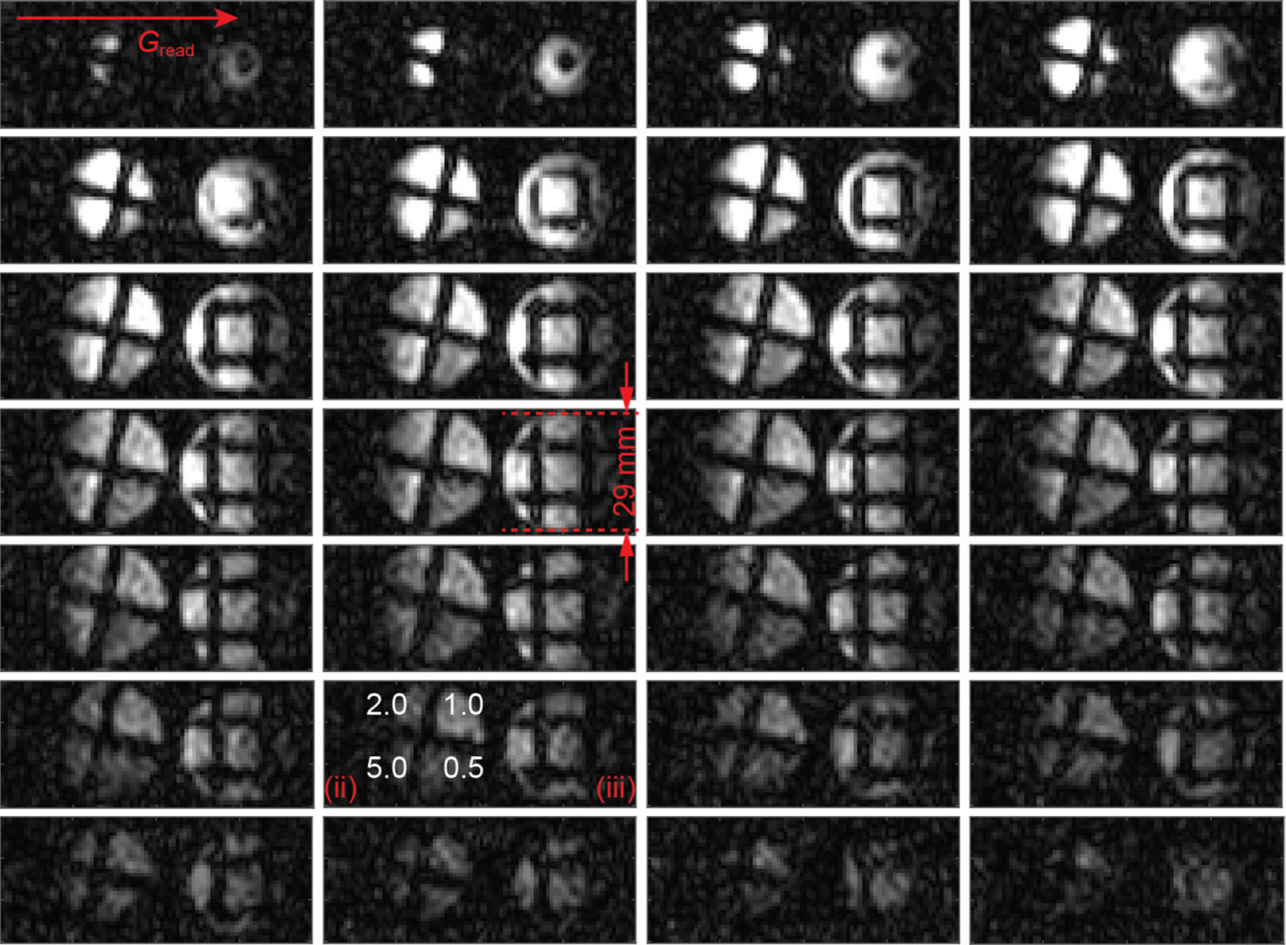
3D bSSFP180 image illustrated as 2D slices of phantom (ii, Figure 2c) (left) and (iii, Figure 2c) (right) acquired at *B*_0_ = 707 μT (RF_ODNP_ frequency ≈ 87 MHz). Slices are ordered from left to right and top to bottom. Sequence parameters are listed in Table 1. The white numbers on phantom (ii) indicate the TEMPO concentration in mM.

Both sequences can be extended with an additional phase‐encoding gradient to enable the acquisition of 3D images. Figure 6 shows the 3D reconstruction obtained using the bSSFP180 sequence for phantoms (ii) and (iii). As with the 2D bSSFP180 results, no banding artifacts are observed [60].

Note that the signal intensity decreases toward the outer borders in the direction of the read gradient, *G*_read_. This decrease is due to the sensitivity profile of the pickup coil of the SQUID‐based magnetic field detector. Although the RF_ODNP_ pulse has a limited bandwidth due to the turned‐on gradients during hyperpolarization, the bandwidth is still sufficiently large to excite the entire sample.

## 4 Discussion

We successfully implemented the bSSFP OMRI sequence, initially introduced by Sarracanie et al. [43], in an 850 μT SQUID‐based ultralow‐field (ULF) MRI system operating at an *RF*_ODNP_ frequency of approximately 90 MHz. This implementation enabled both 2D and 3D MR image acquisition. However, the resulting images displayed pronounced banding artifacts caused by *B*_0_ inhomogeneities, a well‐known limitation of bSSFP sequences in non‐uniform magnetic fields. Simulations estimated a field variation of ≈70 nT (82 ppm) across the phantom (Figure S3). For a repetition time (TR) of 100 ms, signal voids are expected every 117 nT, yet the observed distance between voids in Figure 3a was ≈190 mm, suggesting that the actual *B*_0_ inhomogeneity was roughly twice the simulated value.

For the bSSFP profile calculations (Figure 4), a field inhomogeneity of *σ* = 18.51 ppm (15.73 nT) was assumed after applying first‐order shimming. Experimental bSSFP profile measurements matched the Bloch simulation model qualitatively well. Minor discrepancies in signal amplitude and shape can be attributed to several factors: Despite first‐order shimming, the magnetic field remained slightly inhomogeneous. The signal enhancement was calibrated against an unenhanced thermal equilibrium low‐amplitude reference spectrum, introducing a systematic error. The simulations used idealized hard pulses, while the experimental soft *B*_1_ pulse duration was 5 ms. Especially *B*_0_ field inhomogeneity distribution used in the simulation leads to different patterns within the bSSFP profiles. These types of phase profiles are well‐understood for the classical bSSFP sequences that are routinely used in MRI [7]. The main difference between these bSSFP sequences and the bSSFP OMRI sequence demonstrated here is hyperpolarization of the sample via ODNP during phase encoding. This hyperpolarization period does not alter the shape of the profiles; however, it scales the profiles according to the *E*_max_ value.

To overcome the challenge of *B*_0_ inhomogeneity, we modified the sequence to include two 180‐degree refocusing *B*_1_ pulses, in addition to the standard 90‐degree *B*_1_ pulses for the bSSFP sequence. At low magnetic fields, the lower *B*_1_ frequency required for the 180‐degree pulses results in a negligible SAR [65] making their inclusion viable, even for potential in vivo applications. This is different from high‐field MRI, where such pulses would produce severe SAR issues, making bSSFP with large flip angles unsuitable for in vivo applications. The refocusing pulses effectively stabilize the bSSFP signal with respect to *B*_0_ inhomogeneity, resulting in a new bSSFP180 sequence for hyperpolarized MRI that eliminates banding artifacts.

This study investigated the sequence in combination with non‐selective *B*_1_ pulses. Further investigation is needed on the use of selective *B*_1_ pulses within the sequence.

Results indicate that including 180‐degree pulses decreases the sequence's duty cycle with respect to the RF_ODNP_ field, as the RF_ODNP_ field's duration is limited by the distance between the two 180‐degree pulses. However, this can be compensated for by increasing the RF_ODNP_ amplitude during the hyperpolarization phase. The RF_ODNP_ field is the SAR‐limiting factor for this sequence, so we focused on optimizing it to prevent sample heating and enhance in vivo applications.

The bSSFP180 sequence differs fundamentally from conventional spin‐echo and gradient‐echo (GRE) approaches because it operates in a steady‐state regime instead of producing largely independent echoes. In this steady state, both transverse and longitudinal magnetization from previous excitations are continuously recycled, rather than being fully dephased between repetitions. This results in distinct signal behavior and contrast. This polarization recycling is a key advantage of bSSFP and bSSFP180, leading to higher acquisition efficiency than spin‐echo or GRE‐based methods. As mentioned in the bSSFP study by Sarracanie et al. [54] this approach accelerates imaging approximately by a factor of two compared to previously reported (fast) spin‐echo–based OMRI methods [66] while simultaneously achieving substantially improved spatial resolution.

A further important difference concerns the temporal origin of the measured signal. In spin‐echo–based OMRI sequences, the detected signal reflects the magnetization present at the time of the excitation pulse. In contrast, steady‐state sequences such as bSSFP incorporate magnetization that enters the imaging slice during the readout period. Consequently, SE‐based approaches are often advantageous for dynamic studies, where the signal should correspond to a well‐defined excitation time point, whereas steady‐state sequences can provide higher overall signal due to the continuous recycling and inflow of polarization.

Another fundamental difference between the steady‐state sequences used in OMRI and the conventional SE or GRE sequences is that the readout phase in SE and GRE implementations is independent of the hyperpolarization phase. This separation allows for magnetic field cycling during the hyperpolarization period. Consequently, most OMRI experiments employ field cycling [49], rendering a quantitative SNR comparison at constant magnetic field strength of limited relevance. In practice, field cycling can occur in both directions. MRI systems operating at fields greater than 20 mT typically reduce the magnetic field during OMRI experiments to accommodate the RF_ODNP_ excitation frequency and improve RF penetration depth, which depends on the sample volume. In contrast, the magnetic field is commonly increased during the hyperpolarization phase in the ultralow‐field regime. Due to these fundamental differences in sequence operation, direct quantitative comparisons with conventional spin‐echo or GRE OMRI techniques are not straightforward.

The bSSFP180 sequence offers clear advantages in OMRI settings, particularly in low‐field scanners based on Halbach magnet designs, where field instability and inhomogeneity are significant concerns [67]. Although these systems are gaining traction due to their portability, affordability, and minimal infrastructure requirements [68], they are often affected by temperature‐induced field drift and *B*_0_ field inhomogeneities of several hundred parts per million (ppm). The bSSFP180 sequence offers robustness against *B*_0_ instability, thereby expanding the practicality of OMRI in such systems.

Depending on the chosen spin probe, OMRI can be made sensitive to physiological parameters such as pH and tissue oxygenation [28, 31, 32, 37, 38], underscoring its potential for tumor characterization and other diagnostic applications. Although the availability of stable and biocompatible free radicals remains a limitation, ongoing research is expected to overcome this challenge [46]. Further studies on contrast mechanisms are necessary before applying the new bSSFP180 sequence to such applications. In the ULF regime, where *T*_1_ and *T*_2_ are nearly identical, image contrast primarily reflects proton density. However, the MR signal in both bSSFP and bSSFP180 sequences also depends on *E*_max_, which is influenced by the type and concentration of the radical, RF_ODNP_ power and by tissue‐specific properties. The contrast between the different TEMPO concentrations in phantom (ii) in Figure 6 is barely visible, indicating that proton density is the dominant contrast mechanism at the TR and RF_ODNP_ power used in this experiment. In contrast, a multi–spin‐echo sequence allows the generation of a *T*_2_ map (Figure S6), in which clear contrast between the four TEMPO concentrations can be observed. Another aspect that warrants investigation is the use of long repetition times (TR ≥ *T*_2_) with the bSSFP180 sequence, where *T*_2_ relaxation could contribute additional contrast.

In conclusion, the development of the bSSFP180 sequence represents a significant step forward in enabling artifact‐free imaging in low‐field environments. This sequence combines the advantages of fast and efficient bSSFP imaging with the stability and robustness of spin‐echo sequences. Combined with advances in portable scanner technology and hyperpolarization techniques, this approach has the potential to open new frontiers for accessible, functional [69], and physiologically sensitive imaging in both research and clinical settings.

## Funding

This work was supported by the European Research Council, 834940; Deutsche Forschungsgemeinschaft, 469366436, 527345502, 530130666.

## Supporting information


**Data S1:** Supporting Information.

**Data S2:** Supporting Information.

## Data Availability

The data that supports the findings of this study are available in the Supporting Information of this article.

## References

[1] R. Pohmann , O. Speck , and K. Scheffler , “Signal‐to‐Noise Ratio and MR Tissue Parameters in Human Brain Imaging at 3, 7, and 9.4 Tesla Using Current Receive Coil Arrays,” Magnetic Resonance in Medicine 75 (2016): 801–809. 25820458 10.1002/mrm.25677

[2] M. A. Bernstein , K. F. King , and X. J. Zhou , “Basic Pulse Sequences,” in Handbook of MRI Pulse Sequences (Elsevier, 2004).

[3] J. Eills , D. Budker , S. Cavagnero , et al., “Spin Hyperpolarization in Modern Magnetic Resonance,” Chemical Reviews 123 (2023): 1417–1551. 36701528 10.1021/acs.chemrev.2c00534 PMC9951229

[4] B. M. Dale , M. A. Brown , and R. C. Semelka , MRI Basic Principles and Applications (Wiley, 2015).

[5] K. Scheffler and S. Lehnhardt , “Principles and Applications of Balanced SSFP Techniques,” European Radiology 13 (2003): 2409. 12928954 10.1007/s00330-003-1957-x

[6] O. Bieri and K. Scheffler , “Fundamentals of Balanced Steady State Free Precession MRI,” Journal of Magnetic Resonance Imaging 38 (2013): 2–11. 23633246 10.1002/jmri.24163

[7] C. Ganter , “Static Susceptibility Effects in Balanced SSFP Sequences,” Magnetic Resonance in Medicine 56 (2006): 687. 16826609 10.1002/mrm.20986

[8] F. O. Zelaya , W. U. Roffmann , S. Crozier , S. Teed , D. Gross , and D. M. Doddrell , “Direct Visualisation of B1 Inhomogeneity by Flip Angle Dependency,” Magnetic Resonance Imaging 15 (1997): 497. 9223051 10.1016/s0730-725x(96)00396-7

[9] M. Weigel , “Extended Phase Graphs: Dephasing, RF Pulses, and Echoes ‐ Pure and Simple,” Journal of Magnetic Resonance Imaging 41 (2015): 266–295. 24737382 10.1002/jmri.24619

[10] K. Scheffler , E. Seifritz , D. Bilecen , et al., “Detection of BOLD Changes by Means of a Frequency‐Sensitive trueFISP Technique: Preliminary Results,” NMR in Biomedicine 14 (2001): 490–496. 11746942 10.1002/nbm.726

[11] A. Comment and M. E. Merritt , “Hyperpolarized Magnetic Resonance as a Sensitive Detector of Metabolic Function,” Biochemistry 53 (2014): 7333–7357. 25369537 10.1021/bi501225t PMC4255644

[12] P. Nikolaou , B. M. Goodson , and E. Y. Chekmenev , “NMR Hyperpolarization Techniques for Biomedicine,” Chemistry ‐ A European Journal 21 (2015): 3156–3166. 25470566 10.1002/chem.201405253 PMC4418426

[13] D. A. Barskiy , A. M. Coffey , P. Nikolaou , et al., “NMR Hyperpolarization Techniques of Gases,” Chemistry ‐ A European Journal 23 (2017): 725–751. 27711999 10.1002/chem.201603884 PMC5462469

[14] S. S. Deen , C. Rooney , A. Shinozaki , et al., “Hyperpolarized Carbon 13 MRI: Clinical Applications and Future Directions in Oncology,” Radiology. Imaging Cancer 5 (2023): e230005. 37682052 10.1148/rycan.230005 PMC10546364

[15] P. Dutta , G. V. Martinez , and R. J. Gillies , “A New Horizon of DNP Technology: Application to In‐Vivo 13C Magnetic Resonance Spectroscopy and Imaging,” Biophysical Reviews 5 (2013): 271. 26491489 10.1007/s12551-012-0099-2 PMC4610403

[16] S. J. Nelson , J. Kurhanewicz , D. B. Vigneron , et al., “Metabolic Imaging of Patients With Prostate Cancer Using Hyperpolarized [1‐13C]Pyruvate,” Science Translational Medicine 5 (2013): 198ra108. 10.1126/scitranslmed.3006070 PMC4201045 23946197

[17] T. C. Eisenschmid , R. U. Kirss , P. P. Deutsch , et al., “Para Hydrogen Induced Polarization in Hydrogenation Reactions,” Journal of the American Chemical Society 109 (1987): 8089–8091.

[18] R. W. Adams , J. A. Aguilar , K. D. Atkinson , et al., “Reversible Interactions With Para‐Hydrogen Enhance NMR Sensitivity by Polarization Transfer,” Science 323 (2009): 1708–1711. 19325111 10.1126/science.1168877

[19] A. B. Schmidt , J. Eills , L. Dagys , et al., “Over 20% Carbon‐13 Polarization of Perdeuterated Pyruvate Using Reversible Exchange With Parahydrogen and Spin‐Lock Induced Crossing at 50 μT,” Journal of Physical Chemistry Letters 14 (2023): 5305–5309. 37267594 10.1021/acs.jpclett.3c00707

[20] S. J. McBride , M. Pike , E. Curran , et al., “Scalable Hyperpolarized MRI Enabled by Ace‐SABRE of [1‐13C]Pyruvate,” Angewandte Chemie, International Edition 64 (2025): e202501231. 40268681 10.1002/anie.202501231 PMC12377446

[21] H. de Maissin , P. R. Groß , O. Mohiuddin , et al., “In Vivo Metabolic Imaging of [1‐13C]Pyruvate‐d3 Hyperpolarized by Reversible Exchange With Parahydrogen**,” Angewandte Chemie, International Edition 62 (2023): e202306654. 37439488 10.1002/anie.202306654

[22] K. MacCulloch , A. Browning , D. O. Guarin Bedoya , et al., “Facile Hyperpolarization Chemistry for Molecular Imaging and Metabolic Tracking of [1–13C]Pyruvate In Vivo,” Journal of Magnetic Resonance Open 16‐17 (2023): 100129. 10.1016/j.jmro.2023.100129 PMC10715622 38090022

[23] S. Punwani , P. E. Z. Larson , C. Laustsen , et al., “Consensus Recommendations for Hyperpolarized [1‐13C]Pyruvate MRI Multi‐Center Human Studies,” Magnetic Resonance in Medicine 94 (2025): 1386. 40523079 10.1002/mrm.30570 PMC12236423

[24] M. Vaeggemose , R. F. Schulte , and C. Laustsen , “Comprehensive Literature Review of Hyperpolarized Carbon‐13 MRI: The Road to Clinical Application,” Metabolites 11 (2021): 219. 33916803 10.3390/metabo11040219 PMC8067176

[25] S. Månsson , J. S. Petersson , and K. Scheffler , “Fast Metabolite Mapping in the Pig Heart After Injection of Hyperpolarized 13C‐Pyruvate With Low‐Flip Angle Balanced Steady‐State Free Precession Imaging,” Magnetic Resonance in Medicine 2012 (1894): 68. 10.1002/mrm.24183 22294528

[26] J. Leupold , O. Wieben , S. Månsson , et al., “Fast Chemical Shift Mapping With Multiecho Balanced SSFP,” Magnetic Resonance Materials in Physics, Biology and Medicine 19 (2006): 267–273. 10.1007/s10334-006-0056-9 17119904

[27] J. Leupold , S. Månsson , J. Stefan Petersson , J. Hennig , and O. Wieben , “Fast Multiecho Balanced SSFP Metabolite Mapping of 1H and Hyperpolarized 13C Compounds,” Magnetic Resonance Materials in Physics, Biology and Medicine 22 (2009): 251–256. 10.1007/s10334-009-0169-z 19367422

[28] K. Golman , J. S. Petersson , J. H. Ardenkjær‐Larsen , et al., “Dynamic In Vivo Oxymetry Using Overhauser Enhanced MR Imaging,” Journal of Magnetic Resonance Imaging 12 (2000): 929–938. 11105032 10.1002/1522-2586(200012)12:6<929::aid-jmri17>3.0.co;2-j

[29] D. E. J. Waddington , M. Sarracanie , N. Salameh , F. Herisson , C. Ayata , and M. S. Rosen , “An Overhauser‐Enhanced‐MRI Platform for Dynamic Free Radical Imaging In Vivo,” NMR in Biomedicine 31 (2018): e3896. 29493032 10.1002/nbm.3896

[30] K. Buckenmaier , M. Rudolph , P. Fehling , et al., “Mutual Benefit Achieved by Combining Ultralow‐Field Magnetic Resonance and Hyperpolarizing Techniques,” Review of Scientific Instruments 89 (2018): 125103. 30599552 10.1063/1.5043369

[31] H. Eto , F. Hyodo , N. Kosem , et al., “Redox Imaging of Skeletal Muscle Using In Vivo DNP‐MRI and Its Application to an Animal Model of Local Inflammation,” Free Radical Biology & Medicine 89 (2015): 1097–1104. 26505925 10.1016/j.freeradbiomed.2015.10.418

[32] N. Kosem , T. Naganuma , K. Ichikawa , et al., “Whole‐Body Kinetic Image of a Redox Probe in Mice Using Overhauser‐Enhanced MRI,” Free Radical Biology & Medicine 53 (2012): 328–336. 22579576 10.1016/j.freeradbiomed.2012.04.026

[33] P. Massot , E. Parzy , L. Pourtau , et al., “In Vivo High‐Resolution 3D Overhauser‐Enhanced MRI in Mice at 0.2T,” Contrast Media & Molecular Imaging 7 (2012): 45–50. 22344879 10.1002/cmmi.464

[34] A. W. Overhauser , “Polarization of Nuclei in Metals,” Physical Review 92 (1953): 411–415.

[35] U. L. Günther , “Dynamic Nuclear Hyperpolarization in Liquids,” Topics in Current Chemistry 335 (2013): 23. 22025060 10.1007/128_2011_229

[36] P. Fehling , K. Buckenmaier , S. A. Dobrynin , et al., “The Effects of Nitroxide Structure Upon 1H Overhauser Dynamic Nuclear Polarization Efficacy at Ultralow‐Field,” Journal of Chemical Physics 155 (2021): 144203. 34654311 10.1063/5.0064342

[37] M. C. Krishna , S. English , K. Yamada , et al., “Overhauser Enhanced Magnetic Resonance Imaging for Tumor Oximetry: Coregistration of Tumor Anatomy and Tissue Oxygen Concentration,” Proceedings. National Academy of Sciences. United States of America 99 (2002): 2216. 10.1073/pnas.042671399 PMC122345 11854518

[38] A. A. Gorodetskii , T. D. Eubank , B. Driesschaert , et al., “Development of Multifunctional Overhauser‐Enhanced Magnetic Resonance Imaging for Concurrent In Vivo Mapping of Tumor Interstitial Oxygenation, Acidosis and Inorganic Phosphate Concentration,” Science Reports 9 (2019): 12093. 10.1038/s41598-019-48524-3 PMC6702349 31431629

[39] S. Matsumoto , H. Utsumi , T. Aravalluvan , et al., “Influence of Proton T1 on Oxymetry Using Overhauser Enhanced Magnetic Resonance Imaging,” Magnetic Resonance Medicine 54 (2005): 213. 10.1002/mrm.20564 15968662

[40] K. Yasukawa , A. Hirago , K. Yamada , X. Tun , K. Ohkuma , and H. Utsumi , “In Vivo Redox Imaging of Dextran Sodium Sulfate‐Induced Colitis in Mice Using Overhauser‐Enhanced Magnetic Resonance Imaging,” Free Radical Biology and Medicine 136 (2019): 1. 30928473 10.1016/j.freeradbiomed.2019.03.025

[41] K. Yasukawa , R. Shigemi , T. Kanbe , et al., “In Vivo Imaging of the Intra‐and Extracellular Redox Status in Rat Stomach With Indomethacin‐Induced Gastric Ulcers Using Overhauser‐Enhanced Magnetic Resonance Imaging,” Antioxidants & Redox Signaling 30 (2019): 1147. 29631421 10.1089/ars.2017.7336

[42] H. Deguchi , K. Yasukawa , T. Yamasaki , et al., “Nitroxides Prevent Exacerbation of Indomethacin‐Induced Gastric Damage in Adjuvant Arthritis Rats,” Free Radical Biology and Medicine 51 (2011): 1799. 21906674 10.1016/j.freeradbiomed.2011.08.010

[43] O. V. Efimova , Z. Sun , S. Petryakov , et al., “Variable Radio Frequency Proton‐Electron Double‐Resonance Imaging: Application to pH Mapping of Aqueous Samples,” Journal of Magnetic Resonance 209 (2011): 227–232. 21320790 10.1016/j.jmr.2011.01.011 PMC3065501

[44] V. V. Khramtsov , G. L. Caia , K. Shet , et al., “Variable Field Proton‐Electron Double‐Resonance Imaging: Application to pH Mapping of Aqueous Samples,” Journal of Magnetic Resonance 202 (2010): 267–273. 20007019 10.1016/j.jmr.2009.11.017 PMC2818733

[45] A. Samouilov , O. V. Efimova , A. A. Bobko , et al., “In Vivo Proton‐Electron Double‐Resonance Imaging of Extracellular Tumor pH Using an Advanced Nitroxide Probe,” Analytical Chemistry 86 (2014): 1045. 24372284 10.1021/ac402230h PMC3981471

[46] V. Goncharov , R. O. Jenkins , D. Belinskaia , et al., “Contrast Agents Based on Human Serum Albumin and Nitroxides for 1 H‐MRI and Overhauser‐Enhanced MRI,” International Journal of Molecular Sciences 2024 (2024): 4041. 10.3390/ijms25074041 PMC11012161 38612851

[47] T. Wu , T. S. Rappaport , and C. M. Collins , The human body and millimeter‐wave wireless communication systems: Interactions and implications. IEEE International Conference on Communications (IEEE, 2015), 2423–2429.

[48] F. Hyodo , T. Naganuma , H. Eto , M. Murata , H. Utsumi , and M. Matsuo , “In Vivo Melanoma Imaging Based on Dynamic Nuclear Polarization Enhancement in Melanin Pigment of Living Mice Using In Vivo Dynamic Nuclear Polarization Magnetic Resonance Imaging,” Free Radical Biology and Medicine 134 (2019): 12093. 10.1016/j.freeradbiomed.2019.01.002 30615920

[49] A. Enomoto and K. Ichikawa , “Research and Development of Preclinical Overhauser‐Enhanced Magnetic Resonance Imaging Ayano Enomoto and Kazuhiro Ichikawa Abstract,” Antioxidants & Redox Signaling 37 (2022): 1094–1110. 35369734 10.1089/ars.2022.0038

[50] N. Koonjoo , E. Parzy , P. Massot , et al., “In Vivo Overhauser‐Enhanced MRI of Proteolytic Activity,” Contrast Media & Molecular Imaging 9 (2014): 363–371. 24729587 10.1002/cmmi.1586

[51] A. Rivot , N. Jugniot , S. Jacoutot , et al., “Magnetic Resonance Imaging of Protease‐Mediated Lung Tissue Inflammation and Injury,” ACS Omega 6 (2021): 15012. 34151082 10.1021/acsomega.1c01150 PMC8209802

[52] D. Boudries , P. Massot , E. Parzy , et al., “A System for In Vivo On‐Demand Ultra‐Low Field Overhauser‐Enhanced 3D‐Magnetic Resonance Imaging,” Journal of Magnetic Resonance 348 (2023): 107383. 36724576 10.1016/j.jmr.2023.107383

[53] V. S. Zotev , T. Owens , A. N. Matlashov , I. M. Savukov , J. J. Gomez , and M. A. Espy , “Microtesla MRI With Dynamic Nuclear Polarization,” Journal of Magnetic Resonance 207 (2010): 78–88. 20843715 10.1016/j.jmr.2010.08.015 PMC2956831

[54] M. Sarracanie , B. D. Armstrong , J. Stockmann , and M. S. Rosen , “High Speed 3D Overhauser‐Enhanced MRI Using Combined b‐SSFP and Compressed Sensing,” Magnetic Resonance in Medicine 71 (2014): 735. 23475813 10.1002/mrm.24705

[55] C. Polyon , D. J. Lurie , W. Youngdee , C. Thomas , and I. Thomas , “Field‐Cycled Dynamic Nuclear Polarization (FC‐DNP) of 14N and 15N Nitroxide Radicals at Low Magnetic Field,” Journal of Physics D, Applied Physics 40 (2007): 5527–5532.

[56] K. H. Hausser and D. Stehlik , “Dynamic Nuclear Polarization in Liquids,” Advances in Magnetic and Optical Resonance 3 (1968): 79–139.

[57] C. M. J. Van Uijen and J. H. Den Boef , “Driven‐Equilibrium Radiofrequency Pulses in NMR Imaging,” Magnetic Resonance in Medicine 1 (1984): 502–507. 6571572 10.1002/mrm.1910010409

[58] E. D. Becker , J. A. Ferretti , and T. C. Farrar , “Driven Equilibrium Fourier Transform Spectroscopy. A New Method for Nuclear Magnetic Resonance Signal Enhancement,” Journal of the American Chemical Society 91 (1969): 7784–7785. 5357869 10.1021/ja50001a068

[59] T. Guiberteau and D. Grucker , “{EPR} Spectroscopy by Dynamic Nuclear Polarization in Low Magnetic Field,” Journal of Magnetic Resonance. Series B 110 (1996): 47–54.

[60] R. Graumann , A. Oppelt , and E. Stetter , “Multiple‐Spin‐Echo Imaging With a 2D Fourier Method,” Magnetic Resonance in Medicine 3 (1986): 707–721. 3784888 10.1002/mrm.1910030507

[61] K. L. Seung , M. Mößle , W. Myers , et al., “SQUID‐Detected MRI at 132 μT With T_1_‐Weighted Contrast Established at 10 μT‐300 mT,” Magnetic Resonance in Medicine 53 (2005): 9–14. 15690496 10.1002/mrm.20316

[62] S. A. Srinivas , S. F. Cauley , J. P. Stockmann , et al., “External Dynamic InTerference Estimation and Removal (EDITER) for Low Field MRI,” Magnetic Resonance in Medicine 87 (2022): 614–628. 34480778 10.1002/mrm.28992 PMC8920578

[63] J. Schäper , G. Bauman , C. Ganter , and O. Bieri , “Pure Balanced Steady‐State Free Precession Imaging (Pure bSSFP),” Magnetic Resonance in Medicine 2022 (1886): 87. 10.1002/mrm.29086 PMC9299476 34775622

[64] H. Y. Carr and E. M. Purcell , “Effects of Diffusion on Free Precession in Nuclear Magnetic Resonance Experiments,” Physical Review 94 (1954): 630–638.

[65] J. Parsa and A. Webb , “Specific Absorption Rate (SAR) Simulations for Low‐Field (< 0.1 T) MRI Systems,” Magnetic Resonance Materials in Physics, Biology and Medicine 36 (2023): 429–438. 10.1007/s10334-023-01073-3 PMC10386976 36933091

[66] Z. Sun , H. Li , S. Petryakov , A. Samouilov , and J. L. Zweier , “In Vivo Proton Electron Double Resonance Imaging of Mice With Fast Spin Echo Pulse Sequence,” Journal of Magnetic Resonance Imaging 35 (2012): 471–475. 22147559 10.1002/jmri.22874 PMC3265665

[67] C. Z. Cooley , P. C. McDaniel , J. P. Stockmann , et al., “A Portable Scanner for Magnetic Resonance Imaging of the Brain,” Nature Biomedical Engineering 5 (2021): 229. 10.1038/s41551-020-00641-5 PMC8597947 33230306

[68] B. de Vos , J. Parsa , Z. Abdulrazaq , et al., “Design, Characterisation and Performance of an Improved Portable and Sustainable Low‐Field MRI System,” Frontiers of Physics 9 (2021): 701157.

[69] K. Buckenmaier , A. Pedersen , P. SanGiorgio , K. Scheffler , J. Clarke , and B. Inglis , “Feasibility of Functional MRI at Ultralow Magnetic Field via Changes in Cerebral Blood Volume,” NeuroImage 186 (2019): 185–191. 30394329 10.1016/j.neuroimage.2018.10.071

