# Supplementary material for: A Modified Balanced Steady State Free Precession Sequence for Overhauser Magnetic Resonance Imaging
Source: Magn Reson Med. 2026 Jun 7;96(4):1655–65. doi: 10.1002/mrm.70453 (PMC13418953; doi:10.1002/mrm.70453)
Supplement: Supplementary file 2 — Data S2: Supporting Information. [file MRM-96-1655-s001.pdf]

Supporting information: A modified balanced steady state free precession sequence for Overhauser Magnetic Resonance Imaging

K. Buckenmaier[1,*,§], F. Bullinger[1,§], G. A. Solomakha[1], M. Schneider[1], J. Engelmann[1], P. Pohlmann[2], P. Povolni[1], L. Kuebler[2], A. F. Martins[2] and K. Scheffler[1,3]

[*]corresponding author

[§]shared first authorship

[1] High-Field Magnetic Resonance Center, Max Planck Institute for Biological Cybernetics, Tübingen, 72076, Germany

[2] Werner Siemens Imaging Center, Department of Preclinical Imaging and Radiopharmacy, Eberhard-Karls University; Tübingen 72076, Germany.

[3] Department of Biomedical Magnetic Resonance, Eberhard-Karls University, Tübingen, 72076, Germany

Contents

bSSFP profile Simulations 2

TEMPO structure 4

Image data processing 4

Simulated B_0 field map of the tetracoil 4

Measurement of E_{max} 5

Effect of B_0 inhomogeneity distribution on bSSFP profiles 6

T_2 map of phantom with different TEMPO concentrations 7

bSSFP profile Simulations

The bSSFP profiles are Bloch simulated in the rotating frame. After setting the simulation parameters (Larmor frequency, number of simulated spins per offset angle, number of simulated offset angles, number of sequence repetitions, *TR*, field inhomogeneity, inhomogeneity distribution function, duty cycle, flip angles, relaxation times T_1 and T_2, maximum Overhauser enhancement E_{max}; see table S1), the magnetization is initially set to thermal equilibrium magnetization (M_{II} = 1). B_1 pulses are simulated as rotation matrices around the *x*- or *y*-axis applied to the magnetization vector (green vertical lines in Figure S1). The signs of the two 180°-degree pulses are opposite within one *TR*. B_1 pulse signs are additionally inverted after each *TR*. This is necessary for the experimental realization in order to minimize the effect of imperfect B_1 pulses. Pulses are implemented as instantaneous magnetization flips ("hard" pulses).

During free precession, the transverse magnetization decays with time constant T_2 and longitudinal magnetization with time constant T_1. When hyperpolarization is active (blue area in Figure S1), the Enhancement *E* is set to the Overhauser enhancement $E = E_{ODNP}$ resulting in a magnetization buildup, otherwise it is set to $E = E_{thermal} = 1$. Eq. 1 shows the time evolution after time τ of the magnetization *M* due to relaxation / buildup processes.

$$\begin{pmatrix} M_x(t+\tau) \\ M_y(t+\tau) \\ M_z(t+\tau) \end{pmatrix} = \begin{pmatrix} \exp\left(-\frac{\tau}{T_2}\right) M_x(t) \\ \exp\left(-\frac{\tau}{T_2}\right) M_y(t) \\ E\left(1 - \exp\left(-\frac{\tau}{T_1}\right) \cdot \frac{1-M_z(t)}{E}\right) \end{pmatrix} \qquad \textbf{(eq. 1)}$$

To each simulated spin, a magnetic field inhomogeneity is assigned, which is expressed as a frequency offset $\delta\omega_L$ to the Larmor frequency. The offset angle ϕ was implemented by adding an offset angular velocity around the *z*-axis such that the offset angle was reached after one *TR*. Eq. 2 shows how these respective effects are implemented to the simulation.

$$\begin{pmatrix} M_x(t+\tau) \\ M_y(t+\tau) \\ M_z(t+\tau) \end{pmatrix} = \begin{pmatrix} \cos\left(\left(\frac{\phi}{TR} + \delta\omega_L\right) \cdot \tau\right) & -\sin\left(\left(\frac{\phi}{T_R} + \delta\omega_L\right) \cdot \tau\right) & 0 \\ \sin\left(\left(\frac{\phi}{TR} + \delta\omega_L\right) \cdot \tau\right) & \cos\left(\left(\frac{\phi}{T_R} + \delta\omega_L\right) \cdot \tau\right) & 0 \\ 0 & 0 & 1 \end{pmatrix} \begin{pmatrix} M_x(t) \\ M_y(t) \\ M_z(t) \end{pmatrix} \qquad \textbf{(eq. 2)}$$

The transverse magnetization vector $M_\perp$ is defined as the absolute transverse magnetization, the longitudinal magnetization M_{II} is equal to M_z (eq. 3).

$$M_\parallel = M_z$$
$$M_\perp = |M_x + M_y| \qquad \textbf{(eq. 3)}$$

$M_\perp$ is read out after *TR*/2 (Figure S1) and calculated by the mean value of all simulated spins. Duty cycle is the measure of how long the Overhauser RF field is switched on in relation to *TR*. It is calculated from the exact switching times of the fields in the real experiment.

Figure S1 shows the time evolution of longitudinal and transverse magnetization for a bSSFP (a) and a bSSFP180 (b) sequence. The longitudinal magnetization M_{II} builds up steeply to the enhancement value (~130) during the hyperpolarization phase. During the RF off period it relaxes to thermal equilibrium. Note

that the longitudinal magnetization changes sign after each 180-degree pulse in the bSSFP180 sequence. For the case shown in Figure S1, the steady state is reached after about 20 *TR*s. The number of *TR*s until the steady state is reached is simulation parameter dependent. $M_{\perp}(\phi)$ is read out during the last *TR* of the simulation at *TR*/2 and represents a simulation point within the bSSFP profiles.

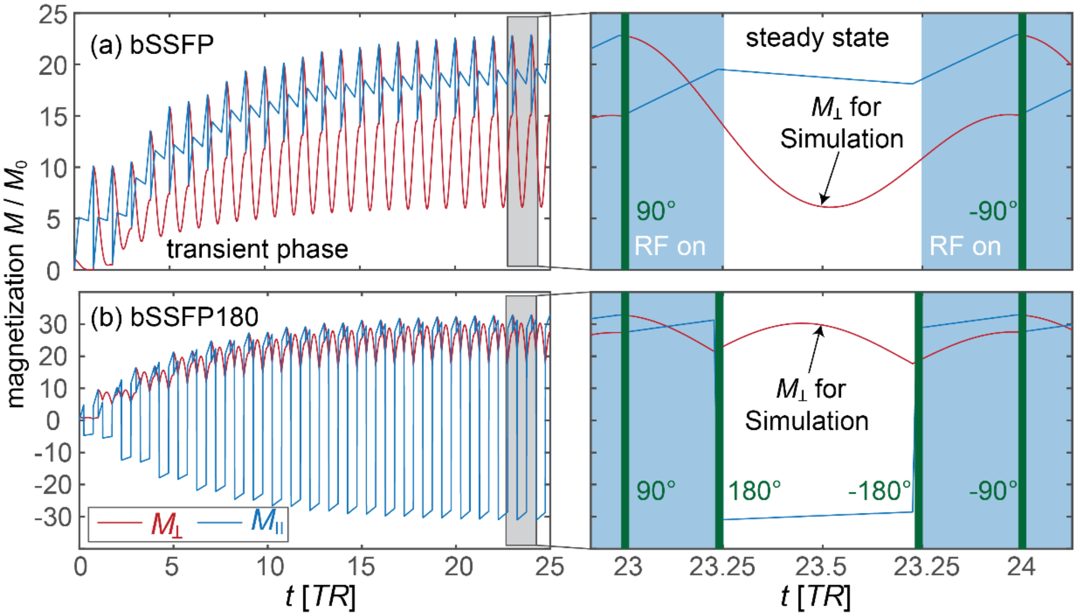

Figure S1: *Simulated qualitative time evolution of transverse,* $M_{\perp}$*, and longitudinal magnetization,* $M_{\parallel}$*, during transient phase (left) and for a zoomed in region within the steady state (right).* $M_{\perp}$ *is read out during the last TR of the simulation at TR/2 and represents a simulation point within the bSSFP profiles.*

Parameter	Brief explanation	Simulation value
numSpins	Number of simulated spins for field inhomogeneity simulation	500
numOffsetAngles	Number of simulated offset angles	361
numTotalRep	Number of simulated repetitions to reach the steady state, last repetition is recorded	50
sweepTR [ms]	Array of simulated *TR*s	[100, 125, 150, 200, 300, 400]
dutycycle_bSSFP	Proportion of time during which Overhauser RF field is turned on for the Overhauser bSSFP sequence for given sweepTR	[0.525, 0.52, 0.517, 0.513, 0.508, 0.506]
dutycycle_bSSFP180	Proportion of time during which Overhauser RF field is turned on for the Overhauser bSSFP180 sequence for given sweepTR	[0.43, 0.444, 0.453, 0.465, 0.477, 0.483]
flip angle alpha [°]	B_1 flip angle alpha	90
f180_1 [°]	First B_1 180° flip angle	180
f180_2 [°]	Second B_1 180° flip angle	180
T1 [ms]	Relaxation time T_1	612.2
T2 [ms]	Relaxation time T_2	563.5

T_ODNP [ms]	Hyperpolarization buildup time constant, (similar to T_1)	612.2
E_ODNP	Overhauser enhancement	132.72
inhomog_ppm [ppm]	B_0 field inhomogeneity in ppm (standard deviation σ of the normal distribution)	18.5
fL [Hz]	Larmor frequency	37100
dfL	Error of the Larmor frequency due to field inhomogeneity	inhomog_ppm × fL / 1E9

Table S1: *Simulation parameters for Figure 4 and 5. The naming of the parameters corresponds to the parameters used in the Matlab script for simulation.*

TEMPO structure

The chemical structure of TEMPO radicals is shown in Figure S2.

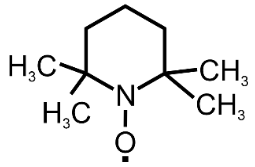

Figure S2: *TEMPO (*2,2,6,6-Tetramethylpiperidinyloxyl) radical

Image data processing

A partial Fourier transform approach was used to reconstruct the MR images by sampling only 50% of the negative *k*-space. This technique exploits the symmetry properties of *k*-space to reduce acquisition time while preserving image quality. In addition, zero-padding with a zero-padding factor of 1 was applied.

Simulated B_0 field map of the tetracoil

Figure S3 shows the simulated B_0 field inhomogeneity of the B_0 tetra coil, which was obtained using the Biot-Savart law. The red circle illustrates the dimensions of the phantom. Only the *yz*-plane is plotted here; however, due to the symmetry of the tetra coil, the inhomogeneity profile is rotationally symmetric around the *z*-axis. The field variation across the phantom is approximately 70 nT (82 ppm).

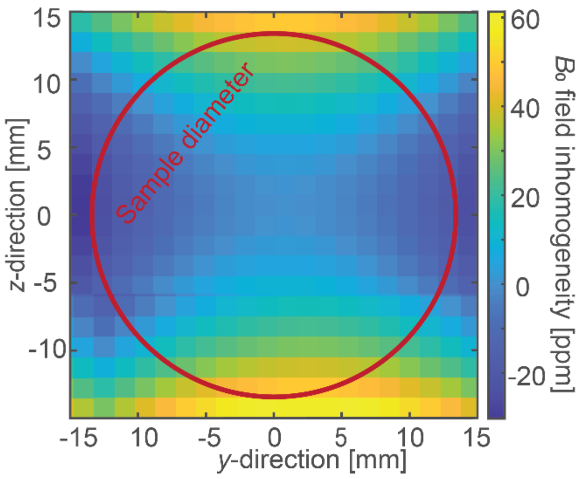

Figure S3: *Simulated B_0 field inhomogeneity in parts per million (ppm) of the tetra coil. The red circle indicates the dimensions of samples (i), (ii) and (iii) from Figure 2c.*

Measurement of E_{max}

Figure S4 shows thermal equilibrium and hyperpolarized spectra. For the hyperpolarized spectrum, the hyperpolarization phase (duration 3.7 s) occurred directly before the free induction decay (FID) read out. The spectra were acquired at B_0 = 870 µT. Data was acquired for 2 s resulting in a frequency resolution of 0.5 Hz. The initial guess for E_{max} = 136, which was used to fit the bSSFP profiles to the experimental data, was obtained by dividing the area under the NMR peak of the hyperpolarized spectrum by the area under the peak of the thermal spectrum.

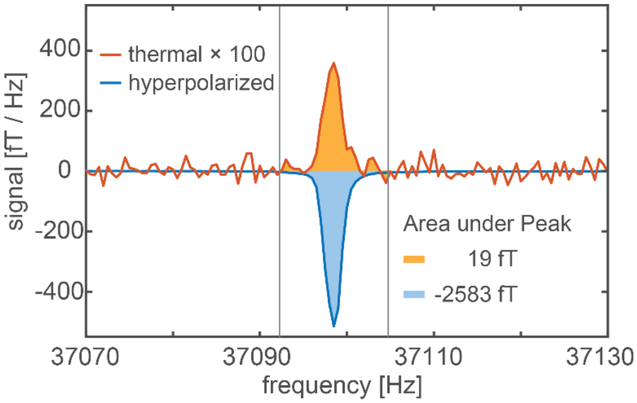

Figure S4: *Measured thermal and hyperpolarized spectra to determine* E_{max}. Grey vertical lines mark the integration interval.

Effect of B_0 inhomogeneity distribution on bSSFP profiles

In Figure S5, the effect of different inhomogeneity distribution functions (left) and their corresponding simulated bSSFP profiles (right) are shown. In Figure S5a), the standard deviation was fit to the measurement data. Adding an offset of 10 ppm in the inhomogeneity distribution shifts the phase profiles to smaller offset angles, but does not break their axial symmetry [Figure S5b)]. Simulating larger inhomogeneity leads to less transverse magnetization in the steady state [Figure S5c)]. This effect is more proncounced for longer TR, where spins dephase more. Simulation parameters can be found in Table S1.

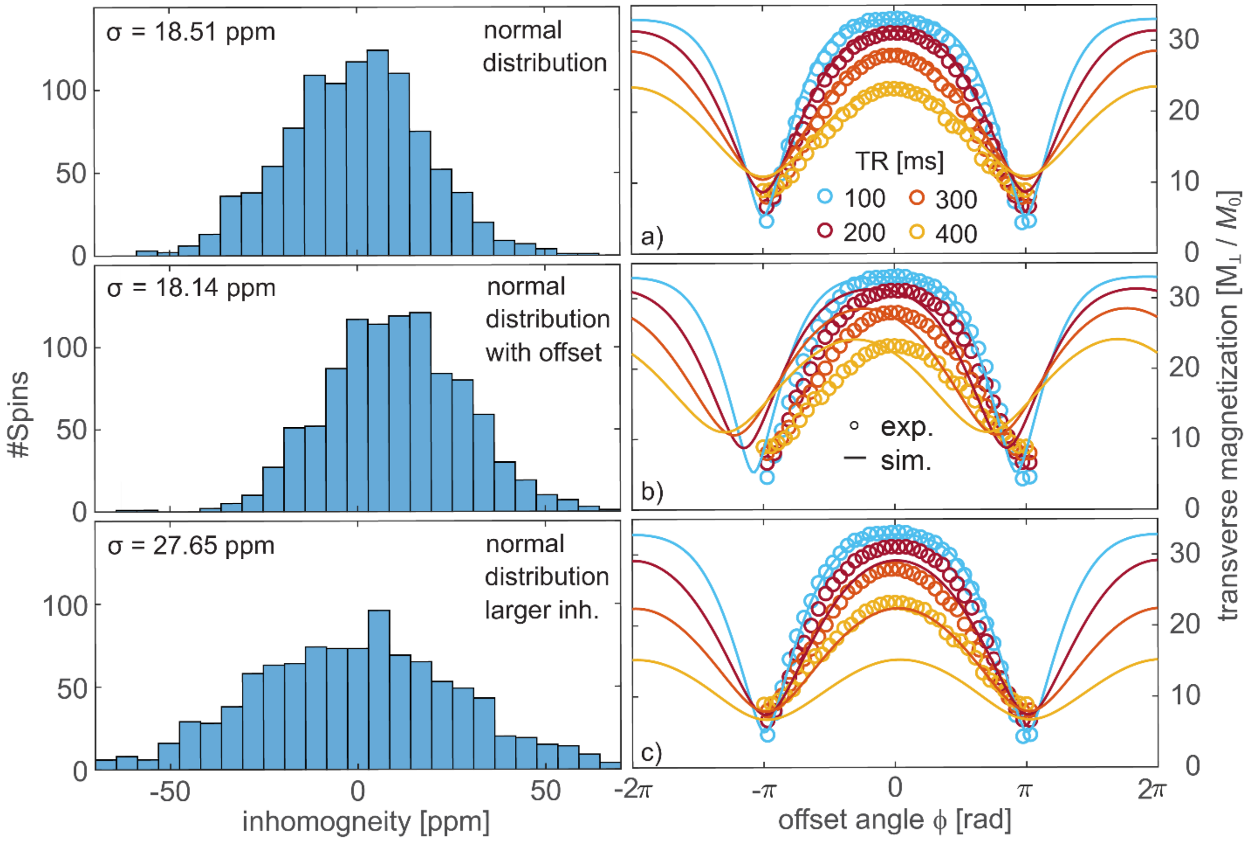

Figure S5: *Different B_0 inhomogeneity distributions (left), were used to simulate respective bSSFP profiles (right).*

T_2 map of phantom with different TEMPO concentrations

The T_2 relaxation map (Figure S6) of the phantom containing different TEMPO concentrations, as well as that of the spherical phantom with a grid insert (Figure 2c, (ii) and (iii)), was acquired using a multiple spin-echo sequence. In this sequence, a hyperpolarization phase was applied prior to each readout phase, consisting of the acquisition of multiple echoes for a single *k*-space line (the sequence parameters are given in Table S2).

The T_2 relaxation map (Figure S6(a)) was generated by fitting the signal intensity of each voxel across the echoes of the multi-spin-echo sequence using a mono-exponential decay function $P(\text{echo}) = ae^{-TE\cdot\text{echo}/T2}$, where P(echo) represents the signal intensity of a voxel at a given echo and *TE* is the echo time. Only voxels with signal intensities above a predefined threshold in the first echo were included in the fitting procedure. The orientation of the phantom is identical to that shown in Figure 6 of the main manuscript.

Additionally, the MR signal within the colour-coded regions of interest (ROIs) was averaged across the echoes and the same mono-exponential decay function was fitted to the averaged ROI signal. The resulting T_2 values, including the corresponding error bars, are shown in Figure S6(b). Note that the error bar for the 0.5 mM concentration is larger than for the other concentrations because the fit includes only the early part of the exponential decay.

A clear contrast between the four chambers of phantom (ii) is evident. The chamber containing 2 mM TEMPO has the same T_2 value as phantom (iii) because the TEMPO concentration in phantom (iii) is also 2 mM.

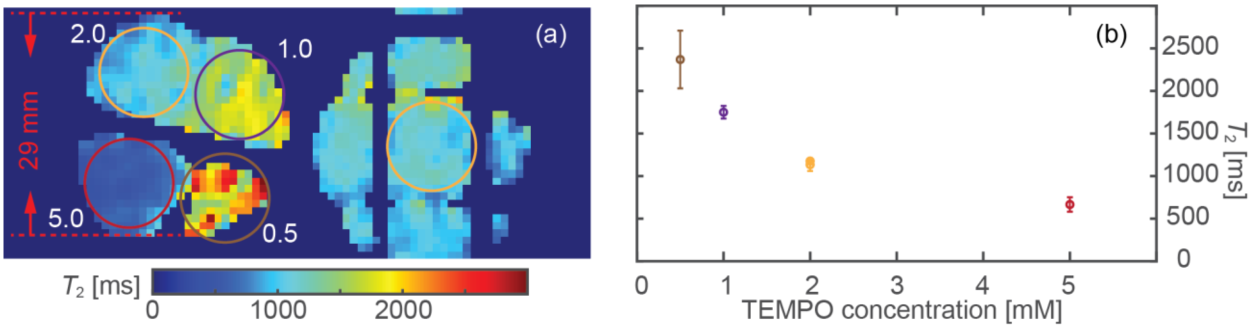

Figure S6: *T_2 relaxation map of phantoms (ii) and (iii) acquired with a multi spin-echo sequence at B_0 = 707 µT (RF_{ODNP} frequency ≈ 87 MHz), as shown in the 3D image of Figure 6 of the main manuscript, (a) together with the corresponding color-coded T_2 values (b) for the ROI indicated by the colored circles in (a). The white numbers in (a) indicate the TEMPO concentrations (mM) in the four chambers of phantom (ii).*

t_{hyp} [ms]	t_{acq} [ms]	Avgs.	# echoes	Phase steps	*TR* [ms]	*TE* [ms]	T_{total}	G_{read} res. [mm]	G_{phase} res. [mm]
900	377	4	8	13	4376	400	3 min 47s	1.8	1.8

Table S2: *Sequence parameters for the T_2 relaxation map of Figure S6.*
